# Glaucomatous Maculopathy: Thickness Differences on Inner and Outer Macular Layers between Ocular Hypertension and Early Primary Open-Angle Glaucoma Using 8 × 8 Posterior Pole Algorithm of SD-OCT

**DOI:** 10.3390/jcm9051503

**Published:** 2020-05-16

**Authors:** Jose Javier Garcia-Medina, Monica del-Rio-Vellosillo, Ana Palazon-Cabanes, Maria Dolores Pinazo-Duran, Vicente Zanon-Moreno, Maria Paz Villegas-Perez

**Affiliations:** 1 Department of Ophthalmology, General University Hospital Morales Meseguer, 30007 Murcia, Spain; 2 Department of Ophthalmology, General University Hospital Reina Sofia, 30003 Murcia, Spain; a.palazoncabanes@gmail.com (A.P.-C.); mpville@um.es (M.P.V.-P.); 3 Department of Ophthalmology and Optometry, University of Murcia, 30120 Murcia, Spain; 4 Ophthalmic Research Unit Santiago Grisolia/FISABIO, 46017 Valencia, Spain; 5 Cellular and Molecular Ophthalmolobiology Group, Surgery Department of the University of Valencia, 46010 Valencia, Spain; 6 Red Temática de Investigación Cooperativa en Patología Ocular (OFTARED), Instituto de Salud Carlos III, 28029 Madrid, Spain; 7 Department of Anesthesiology, University Hospital Virgen de la Arrixaca, 30120 Murcia, Spain; monicadelriov@hotmail.com; 8 Area of Health Sciences, Valencian International University, 46002 Valencia, Spain; vczanon@universidadviu.com

**Keywords:** glaucoma, ocular hypertension, optical coherence tomography, posterior pole algorithm, macula, layer, heatmap, Spectralis, outer retina, inner retina

## Abstract

The purpose of this study was to compare the thickness of all inner and outer macular layers between ocular hypertension (OHT) and early primary open-angle glaucoma (POAG) using spectral domain optical coherence tomography (SD-OCT) 8 × 8 posterior pole algorithm (8 × 8 PPA). Fifty-seven eyes of 57 OHT individuals and fifty-seven eyes of 57 early POAG patients were included. The thickness of macular retinal nerve fiber layer (mRNFL), ganglion cell layer (GCL), inner plexiform layer (IPL), inner nuclear layer (INL), outer plexiform and nuclear layer, photoreceptor layer (PRL) and retinal pigment epithelium were obtained in 64 cells for each macular layer and mean thickness of superior and inferior hemispheres was also calculated. Thinning of superior and inferior hemisphere mean thickness in mRNFL, GCL and IPL and thickening of superior and inferior hemisphere mean thickness in PRL and inferior hemisphere in INL were found in early GPAA group. Otherwise, heatmaps representing cell-to-cell comparisons showed thinning patterns in inner retinal layers (except for INL) and thickening patterns in outer retinal layers in GPAA group. We found that 8 × 8 PPA not only allows the detection of significant thinning patterns in inner retinal layers, but also thickening patterns in outer retinal layers when comparing early POAG eyes to OHT eyes.

## 1. Introduction

Glaucoma is one of the most frequent causes of blindness worldwide and its incidence is increasing [1]. The commonest type of glaucoma affecting Caucasian population is primary open-angle glaucoma (POAG). POAG is characterized by apoptosis of retinal ganglion cells (RGCs) producing a progressive and characteristic visual field loss [2]. Ocular hypertension (OHT) is a pre-disease state in which the intraocular pressure (IOP) is high according to standards but has not damaged the RGCs. A key point in the management of OHT or POAG is the moment to introduce medical therapy, because it is usually a lifelong course of treatment. This decision is normally based on different clinical signs, but in our actual daily practice, most times the decision is made on the basis of the optical coherence tomography (OCT) findings. Thus, it is important to investigate the OCT differences of retinal structure between OHT and early POAG [3].

At present, spectral-domain OCT (SD-OCT) is considered one of the most important ancillary tests in the diagnosis and follow-up of POAG. With this technology papillary, peripapillary and macular regions are usually assessed to investigate RGC integrity when dealing with glaucoma patients. Macular region contains more than half of RGC of the eye [4] and is anatomically less variable than papillary and peripapillary locations. Thus, macular region has been considered an optimal location to study glaucomatous changes when there is not a concurrent macular disease [5]. However, RGCs are only one type of cells integrated in the retinal circuitry, which is organised in several layers [6].

In the last few years, SD-OCT technology has launched protocols that allow the automatic measurement of the thickness of all individual macular layers. Furthermore, a number of maps or algorithms have emerged to represent these macular thicknesses: Early Treatment Diabetic Retinopathy Study (ETDRS) grid, elliptical map, 7 mm × 7 mm map or 8 × 8 posterior pole algorithm (8 × 8 PPA) [5].

All authors agree that there is thinning of the inner retinal layers in POAG. However, controversy exists concerning the behaviour of outer retinal layers in this disease [7,8,9,10,11,12,13,14,15,16,17].

In this study we aimed to compare the topographical differences in thickness of inner and outer macular layers between OHT and early POAG patients using 8 × 8 PPA. As far as we know, this is the first study performed in this sense with this algorithm.

## 2. Materials and Methods

This prospective, cross-sectional study recruited participants between January and October 2017 at the Department of Ophthalmology, University Hospital Reina Sofía Murcia, Spain. All patients signed informed consent. The investigation was approved by the institutional review board of the University Hospital Reina Sofía Murcia (approval number 12/16) and adhered to the Helsinki declaration.

All participants had a complete ophthalmic examination including: autorefractometry, best-corrected visual acuity (BCVA), standard automated perimetry, SD-OCT of the macula, IOP by applanation tonometry, biomicroscopy, central corneal thickness, gonioscopy and dilated fundus exploration.

The real IOP value obtained by applanation tonometry may vary according to central corneal thickness. Thus, IOP in this study was adjusted by corneal pachymetry [18]. The number of antiglaucoma eyedrops was also considered. Only one eye was selected per patient. When both eyes were eligible, one of them was randomly chosen.

The inclusion criteria were: Caucasian origin, OHT or early POAG diagnosis, BCVA ≥ 0.5, refractive error of less than 6 dioptres of sphere and 2.5 cylindrical dioptres, with no clinically significant media opacity and with open angle using a goniolens.

OHT patients were defined as having an IOP above 21 mmHg without treatment, normal standard automated perimetry, with no signs of glaucomatous damage in the optic nerve. Early POAG patients were defined as having signs suggestive of optic nerve glaucomatous damage (cupping, notching or haemorrhage), glaucomatous defects in standard automated perimetry (see below), and mean deviation ≤ −6 decibels according to Hodapp classification [19].

The exclusion criteria were: ocular disease other than OHT or POAG, ocular surgery in the previous year, unreliable perimetry or OCT examinations or the presence of systemic or neurological diseases that might had interfered with perimetry or OCT performance (e.g., Alzheimer’s or Parkinson’s disease).

Standard automatic perimetry assessments were performed in both eyes using the SITA fast strategy and 30-2 pattern by means of Humphrey Field Analyzer II (Carl Zeiss Meditec, Dublin, CA, USA). Glaucomatous defects in perimetry were: hemifield test outside normal limits or a pattern standard deviation with a probability of <5% or ≥ 3 adjacent points with a significant level <5% with one of these of <1%. Perimetries with false positives or negatives exceeding 33% or the fixation losses exceeding 20% were considered unreliable.

**Figure 1:**
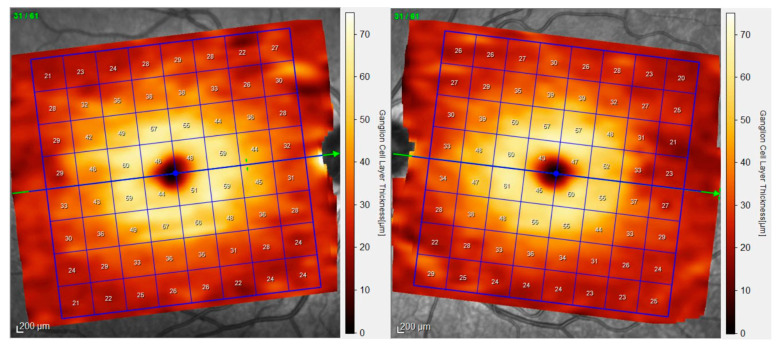
8 × 8 Posterior Pole Algorithm (8 × 8 PPA). This algorithm is made up of 64 cells or superpixels 3 degrees wide. Automatic alignment of the horizontal meridian according to fovea-disc axis induces a slightly rotation of the grids of the right and left eye. The numbers inside the cells indicate mean thickness in microns. In this example, ganglion cell layer is represented.

All patients underwent OCT examinations using 8 × 8 PPA (Spectralis, Heidelberg Engineering, Heidelberg, Germany; 6.0 software version). This algorithm displays the thickness values in microns of the selected layer segmented in an 8 × 8 mm grid centered on the fovea, thus resulting in 64 cells or superpixels 3 degrees wide. Automatic alignment of the horizontal meridian according to fovea-disc axis is provided in 8 × 8 PPA (Figure 1).

**Figure 2:**
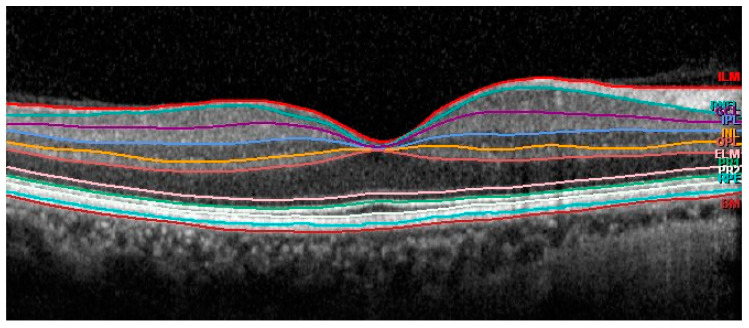
Automatic segmentation of the different retinal layers in macular area.

Thickness data were automatically obtained for the following segmentations: retinal pigment epithelium (RPE) layer, outer retina (including RPE and photoreceptors), outer nuclear layer (ONL), outer plexiform layer (OPL), inner nuclear layer (INL), inner plexiform layer (IPL), ganglion cell layer(GCL) and retina nerve fiber layer (mRNFL) (Figure 2).

**Figure 3:**
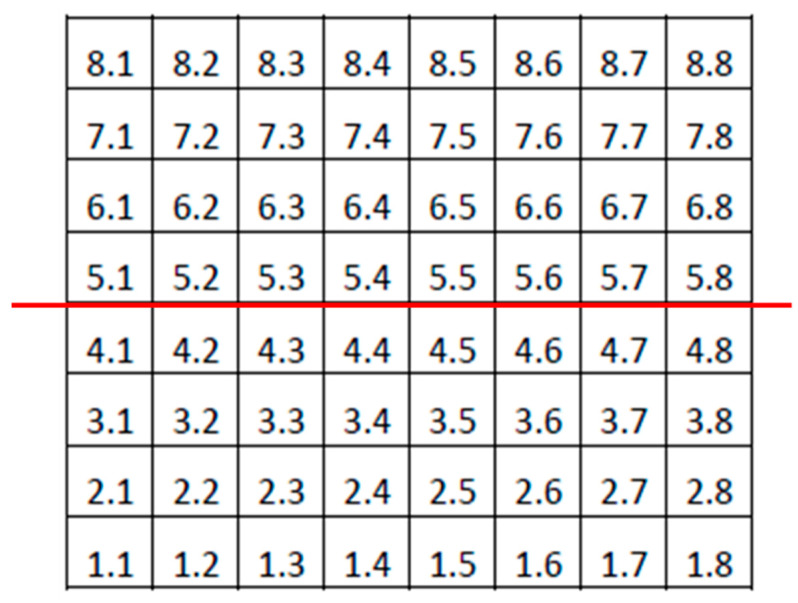
Denomination of cells or superpixels in the 8 × 8 Posterior Pole Algorithm (right eye). The horizontal red line divides the grid into superior and inferior hemispheres. Mean thicknesses of both hemispheres were calculated in this study by averaging the thickness values of the superpixels in each hemisphere.

Only examinations obtaining signal strength ≥ 20 were considered reliable. The same experienced ophthalmologist (J.J.G.M.) checked all the scans. If segmentation errors, decentrations or other artefacts were detected examinations were considered unreliable. ONL and OPL thickness values were added (OPL and ONL) in order to avoid artefactual results due to the orientation of the Henle fibres [20]. Photoreceptor thickness values were calculated by subtracting OPL and ONL thickness from outer retina thickness. In addition, the mean values of the thickness of the superior and inferior hemispheres were also obtained for each layer (Figure 3).

Statistical calculations were performed using SPSS software (IBM, Chicago, IL, USA, version 24). Sex and laterality of the eye were compared using Fisher’s test between groups. All the continuous variables were assessed for normality with the Kolmogorov–Smirnov test and showed a normal distribution except for the number of antiglaucoma eyedrops. Thus, comparisons of mean age, BCVA, IOP, vertical cupping, MD, PSD, hemisphere thickness and the thickness of each superpixel were calculated between groups using Student’s t-tests for the independent samples. The number of antiglaucoma eyedrops was compared using a Mann–Whitney U test. The differences of mean thickness in each superpixel between OHT and early POAG groups were represented using heatmaps (OriginPro 2019 b software, Origin Lab, Northampton, USA). All heatmaps were constructed as if all eyes were right eyes. A color code was used to represent differences in superpixels between the two groups:

Red for thickening and blue for thinning in early POAG group. The color intensity was proportional to the differences in thickness, corresponding more intense blue or red to higher differences in microns. Significant differences in thickness were also represented for all superpixels. Black color was used when no statistically significant differences were detected. Thus, we plotted heatmaps of simple differences to indicate the general trend and heatmap of statistically significant differences. The significance level was *p* < 0.05.

## 3. Results

Fifty-seven eyes of 57 OHT patients and fifty-seven eyes of 57 early POAG patients were finally included. The demographic and clinical data are presented in Table 1.

The mean thickness of the different retinal layers measured in the superior and inferior hemisphere can be observed in Table 2. When we compared these values between the groups we found a significant decreases in the thickness of the mRNFL, GCL and IPL in both the superior and inferior hemispheres, and significant increases in the thickness of the INL (inferior hemisphere) and PRL (superior and inferior hemisphere) in the POAG group (Table 2).

**Figure 4:**
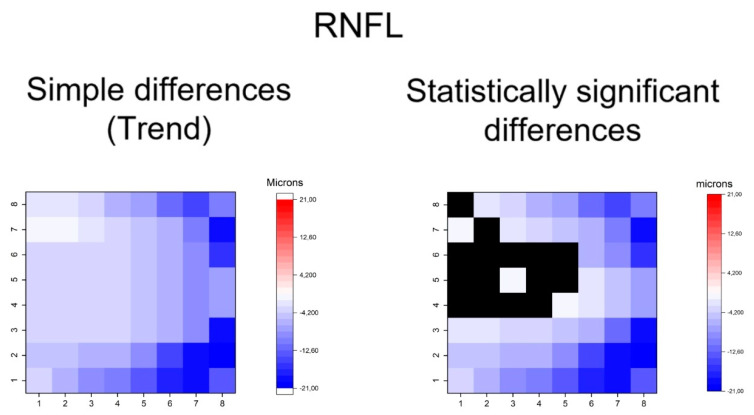
Heatmaps showing differences in macular retinal nerve fiber layer (mRNFL). Left map: this heatmap represents simple differences of mean thickness between OHT group and early POAG group in each superpixel giving an idea of the trend. Right map: the heatmap represents statistically significant differences (*p* < 0.05) of the mean thickness between groups in each superpixel. No significant difference (*p* ≥ 0.05) is represented in black. Range of significant OHT-POAG differences in superpixels between −20.70 and −1.35 microns.

**Figure 5:**
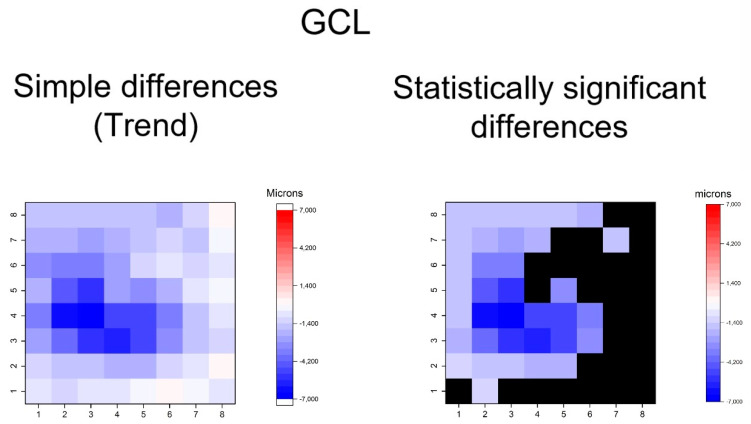
Heatmaps showing differences in ganglion cell layer (GCL). Left map: this heatmap represents simple differences of mean thickness between OHT group and early POAG group in each superpixel, giving an idea of the trend. Right map: the heatmap represents statistically significant differences (*p* < 0.05) of the mean thickness between groups in each superpixel. No significant difference (*p* ≥ 0.05) is represented in black. Range of significant OHT-POAG differences in superpixels between −6.85 and −1.35 microns.

**Figure 6:**
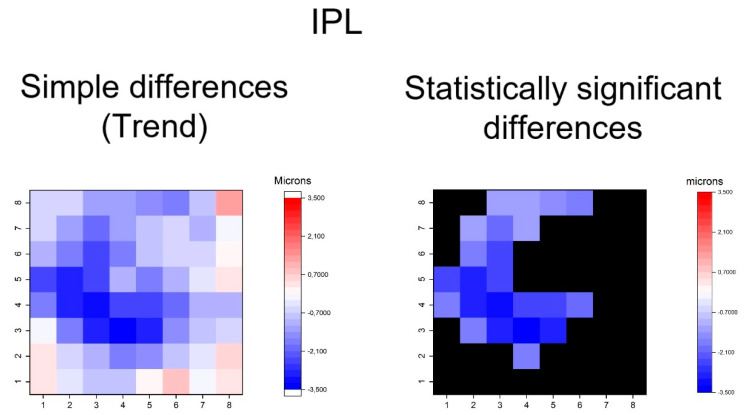
Heatmaps showing differences in inner plexiform layer (IPL). Left map: this heatmap represents simple differences of mean thickness between OHT group and early POAG group in each superpixel giving an idea of the trend. Right map: the heatmap represents statistically significant differences (*p* < 0.05) of the mean thickness between groups in each superpixel. No significant difference (*p* ≥ 0.05) is represented in black. Range of significant OHT-POAG differences in superpixels between −3.36 and −1.22 microns.

**Figure 7:**
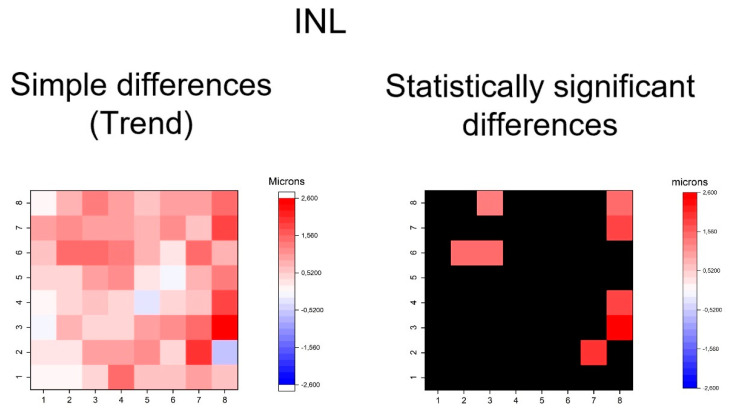
Heatmaps showing differences in inner nuclear layer (INL). Left map: this heatmap represents simple differences of mean thickness between OHT group and early POAG group in each superpixel giving an idea of the trend. Right map: the heatmap represents statistically significant differences (*p* < 0.05) of the mean thickness between groups in each superpixel. No significant difference (*p* ≥ 0.05) is represented in black. Range of significant OHT-POAG differences in superpixels between 1.33 and 2.52 microns.

**Figure 8:**
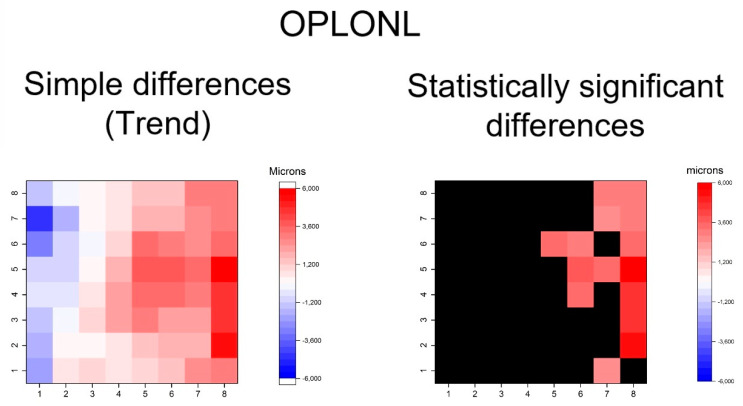
Heatmaps showing differences in outer plexiform and outer nuclear segmentation (OPLONL). Left map: this heatmap represents simple differences of mean thickness between OHT group and early POAG group in each superpixel giving an idea of the trend. Right map: the heatmap represents statistically significant differences (*p* < 0.05) of the mean thickness between the groups in each superpixel. No significant difference (*p* ≥ 0.05) is represented in black. The range of significant OHT-POAG differences in superpixels is between 2.68 and 5.66 microns.

**Figure 9:**
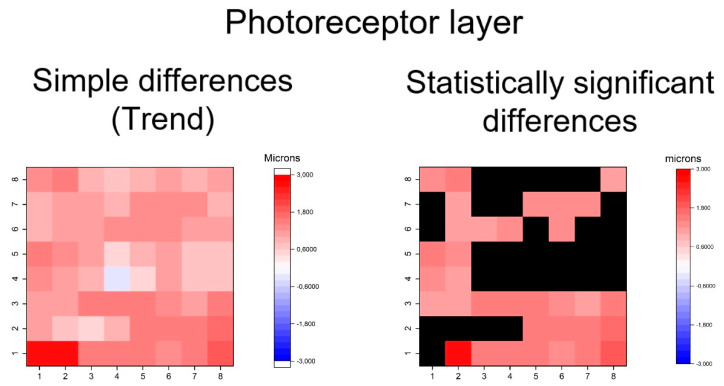
Heatmaps showing differences in photoreceptor layer. Left map: this heatmap represents simple differences in the mean thickness between the OHT group and early POAG group in each superpixel, giving an idea of the trend. Right map: the heatmap represents the statistically significant differences (*p* < 0.05) of the mean thickness between groups in each superpixel. No significant difference (*p* ≥ 0.05) is represented in black. Range of significant OHT-POAG differences in superpixels between 1.08 and 2.63 microns.

**Figure 10:**
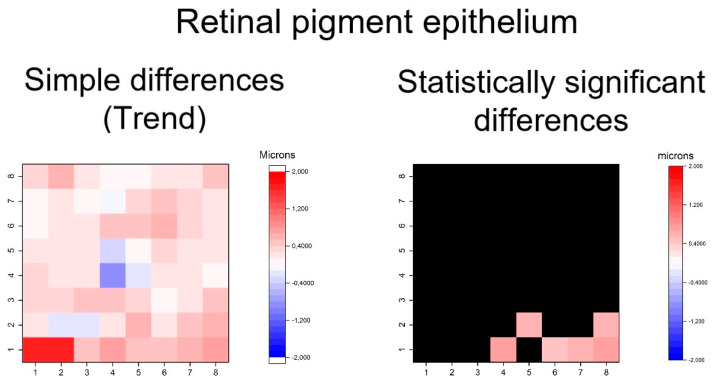
Heatmaps showing differences in the retinal pigment epithelium layer. Left map: this heatmap represents simple differences of mean thickness between OHT group and early POAG group in each superpixel giving an idea of the trend. Right map: the heatmap represents statistically significant differences (*p* < 0.05) of the mean thickness between groups in each superpixel. No significant difference (*p* ≥ 0.05) is represented in black. Range of significant OHT-POAG differences in superpixels between 0.51 and 0.75 microns.

The heatmaps showing the differences in thickness of the different retinal layers measured between the groups can be observed in Figure 4, Figure 5, Figure 6, Figure 7, Figure 8, Figure 9 and Figure 10. In general, heatmaps documented thinning of the inner retinal layers (except for INL) in the early POAG group. The mRNFL showed a symmetrical pattern of peripheral thinning superonasally and inferonasally (Figure 4). GCL (Figure 5) and IPL (Figure 6) exhibited a paracentral temporal and inferior thinning. INL showed a general trend to thickening, with few significant differences in superpixels (Figure 7).

Furthermore, thickening patters in outer retinal layers was exhibited in GPAA group, especially in the OPL and ONL and photoreceptor layers (Figure 8, Figure 9 and Figure 10). The OPL and ONL layers had fewer thickened superpixels, but quantitatively more affected superpixels (in microns) than the photoreceptors layer. In fact, the superpixels in OPLPNL presented differences of up to 6 microns. The OPL and ONL layer was predominantly affected at the nasal superpixels, whereas the photoreceptors layer presents more diffused thickening, predominantly at the inferior and temporal superpixels (Figure 8 and Figure 9). RPE had a similar general trend of thickening, but only few inferior superpixels were significantly affected (Figure 10).

In summary, the inner retina layers showed more accentuated and extensive changes, but outer retinal layers also presented differences. As can be seen, the RNFL and GCL graphical maps display the greatest differences in microns between groups (Figure 4, Figure 5, Figure 6, Figure 7, Figure 8, Figure 9 and Figure 10).

## 4. Discussion

This investigation, in which we compare the thickness of the macular layers in the superpixels of the 8 × 8 PPA between OHT and early POAG patients, showed significant thinning patterns of the inner retinal layers and significant thickening patterns of the outer retinal layers. These changes are more accentuated in inner retina, suggesting that the outer retina is less vulnerable to glaucomatous damage.

Some works have investigated 8 × 8 PPA in glaucoma. Various authors have studied the total macular thickness in glaucomatous patients with this algorithm, but they did not consider individual layer thickness [21,22,23]. Another study compared 8 × 8 PPA data between healthy controls (but not OHT individuals) and POAG patients (bringing together in one group early, moderate and severe glaucoma) but the authors used the mean quadrant thickness (the average of 16 superpixels of each quadrant) for analysis. Thus, they obtained superotemporal, inferotemporal, superonasal and inferonasal thickness values and they only considered inner retinal layers [24].

In a previous study of our group in which we compared the thickness of the different retinal layers using the superpixels of the 8 × 8 PPA between healthy subjects and patients with early, moderate and severe POAG (together in glaucoma group), we found topographical patterns of thinning of the mRNFL, GCL and IPL, and thickening of the INL and outer retinal layers. In the present study, we also document the thinning of the inner retinal layers when we compare OHT with early POAG patients. However, the thickening of the outer retinal layers found in this study affects fewer superpixels than affected in our previous study. However, in general, our present results corroborated the results found in our previous study, although in a different type of patients [16].

Furthermore, a multicentre investigation only considered the average of the 16 most central superpixels of 8 × 8 PPA of inner retinal layers (not outer layers) and compared results between healthy (but not OHT individuals) and all types of POAG patients (but not early POAG patients), and found significant thinning of inner retinal layers of the macula in glaucoma [25].

In a recent study [12], the mean thickness of the superior and inferior hemispheres obtained by 8 × 8 PPA were compared between 15 healthy controls and 15 advanced POAG patients. The authors concluded that outer retinal layers (OPL, ONL and outer retinal layer) were not different between groups. However, the small sample size (n = 15 per group) should be taken into account when interpreting the non-significant results of this study, because the statistical power may be low. Furthermore, the authors considered OPL and ONL individually, so segmentation artifacts may be present when not considering the Henle fiber effect [20].

All these mentioned studies dealing with 8 × 8 PPA in glaucoma, except ours [16], aggregated data and did not consider individual superpixels for analysis. In our opinion, the aggregation of data makes these models lose details when trying to detect subtle changes as those of outer macular layers. That is probably the reason for the difference of the results when considering hemispheres (aggregated data) and individual superpixels (non-aggregated data). We believe that topographical representation using superpixel-to-superpixel heatmaps, as performed in the present study, is a more accurate method for tackling this problem.

Furthermore, previous studies compared healthy controls, but not OHT patients, with glaucoma patients (although most of these studies the glaucoma group included early, moderate and severe glaucomas). However, in our daily clinical practice, one of the main dilemmas that we face is to discriminate between OHT and early POAG in order to consider the start of ocular therapy. The fact that these two evolutionary stages (OHT and early POAG) are so close may justify that the differences between groups were less intense and extensive in the present study than they were in our previous study [16].

**Figure 11:**
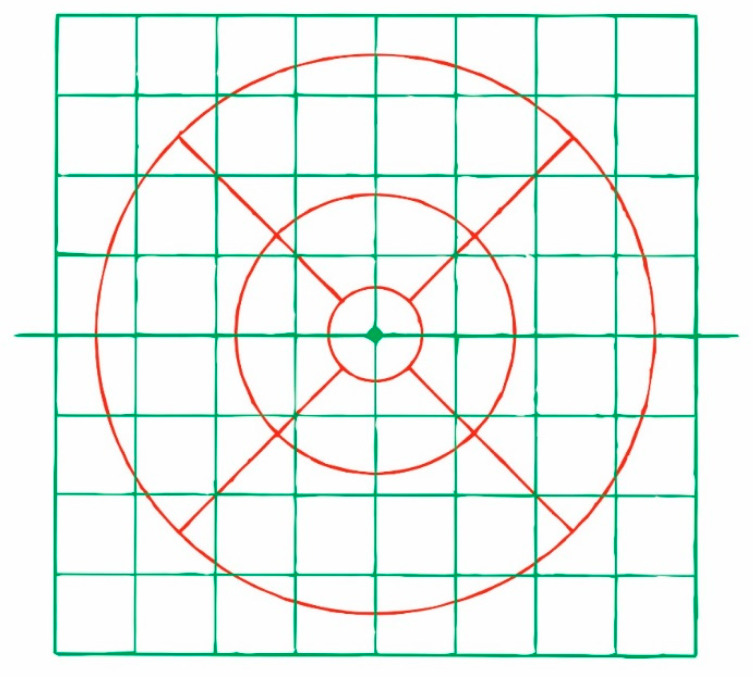
Superposition in real size of the 8 × 8 PPA (green) and ETDRS grid (red).

Other studies have used the ETDRS grid to compare OHT individuals with early POAG patients, and have also demonstrated the thinning of the inner macular layers in glaucoma [11,26,27]. In one of these, the authors also considered outer macular layers and found no thickness differences between groups [11]. However, in our opinion, the ETDRS grid is a suboptimal algorithm to investigate the retinal layer thickness in glaucoma when compared with the 8 × 8 PPA (Figure 11). First, temporal and nasal subfields of the ETDRS grid are crossed by the horizontal midline and, as it is known, glaucomatous damage of the retina is usually different on both sides of the horizontal raphe. Second, the 8 × 8 PPA is wider than the EDTRS grid, allowing us to detect more peripheric changes in posterior pole. Third, the 8 × 8 PPA collects more detailed information than the EDTRS grid because several superpixels are comprised in one ETDRS subfield. Fourth, the ETDRS grid does not take into account the inclination of the fovea-disc axis as the 8 × 8 PPA does (Figure 1). It has been suggested that rotated scans are more accurate than unrotated scans [28], especially in large fovea-disc angles [29].

An important finding of this study is the thickening of the outer macular layers in glaucoma. Previous studies on this subject have found controversial results. Some showed no thickness changes [7,8,9,10,11,12,13], while others found significant thickening of the outer macular layers consistent with the results of this work [14,15,16,17]. This disparity of results may be attributable to differences on the methods used (for example, different OCT algorithms, the 8 × 8 PPA or the ETDRS grid, as discussed above) or to the fact that glaucomatous changes in outer retina layers are much more subtle in the outer retinal layer than in the inner retinal layer and thus require a larger sample size to be detected. Other influencing factors in these dissimilar results may be differences of epidemiological variables such as ethnic background, age and others, or glaucoma severity between the studies.

Our results showing outer retinal thickening in POAG agree with the results of a study carried out in a nonhuman primate model of glaucoma. In this investigation, the authors attributed this phenomenon to the secondary spread of the outer macular layers in order to fill the void of the inner macular layers due to the “scaffold effect” of Müller cells [30]. Other authors have proposed other mechanisms to explain these thickenings, such as photoreceptor swelling, glial cell response, inflammatory phenomena or extracellular remodeling [15,31]. It is remarkable that we could find changes not only in the inner—but also in the outer—macular layers in an early stage of POAG.

The present investigation is limited by a number of facts. First, it is a cross-sectional study, so it does not allow the investigation of progressive changes in the evolution of POAG. Thus, longitudinal studies would be needed to evaluate this aspect. Moreover, the recruited participants were all Caucasian individuals and only one type of glaucoma (POAG) was included; therefore, these results might not be extrapolated to other ethnic groups or to other types of glaucoma.

In conclusion, the 8 × 8 PPA of the SD-OCT allows the detection of significant thinning of the inner retinal layers and thickening of the outer retinal layers when comparing early POAG eyes to OHT eyes.

## Figures and Tables

**Table 1:** Demographics and clinical data of the study groups.

	OHT Group (*n* = 57)	Early POAG Group (*n* = 57)	*p*
Sex (male/female)	23/34	22/35	1 **‡**
Age (years)	65.71 ± 11.80	70.68 ± 11.81	0.127
Right eye/Left eye	28/29	25/32	0.782 **‡**
BCVA (decimal)	0.95 ± 0.24	0.91 ± 0.46	0.210
IOP (mmHg)	18.74 ± 3.76	18.09 ± 3.64	0.352
Vertical cupping	0.35 ± 0.20	0.51 ± 0.26	**<0.001**
MD (dB)	−0.96 ± 0.51	−3.39 ± 1.59	**0.013**
PSD (dB)	1.05 ± 0.52	3.62 ± 2.06	**0.009**
Number of antiglaucoma eyedrops	1.01 ± 0.86	1.46 ± 0.88	**0.006 §**

The values represent mean ± standard deviation. Significant results are indicated in bold. **‡** Fisher test. **§** Mann–Whitney U test. The rest of the *p* values were obtained through an unpaired Student *t*-test. OHT = ocular hypertension; POAG = primary open-angle glaucoma; BCVA = best-corrected visual acuity; IOP = intraocular pressure; MD = mean deviation; PSD = pattern standard deviation.

**Table 2:** Comparisons of mean thickness of superior and inferior hemispheres between OHT and early POAG groups.

Layer	Hemisphere	OHT (Microns)	Early POAG (Microns)	Difference of Means (Microns)	*p*
mRNFL	Superior	42.58 ± 7.19	35.11 ± 7.98	7.47	**<0.001**
	Inferior	37.25 ± 7.43	32.04 ± 5.72	5.21	**<0.001**
GCL	Superior	30.98 ± 2.94	28.75 ± 3.65	2.23	**0.001**
	Inferior	30.68 ± 3.17	28.97 ± 3.54	1.71	**0.09**
IPL	Superior	25.67 ± 2.34	24.57 ± 2.40	1.10	**0.016**
	Inferior	26.25 ± 2.38	25.25 ± 2.38	0.99	**0.032**
INL	Superior	30.60 ± 2.32	31.49 ± 2.45	−0.88	0.055
	Inferior	30.43 ± 2.52	31.53 ± 2.67	−1.09	**0.029**
OPL and ONL	Superior	76.58 ± 4.93	78.07 ± 6.46	−1.49	0.169
	Inferior	81.23 ± 5.08	82.41 ± 6.54	−1.17	0.286
PRL	Superior	75.95 ± 2.52	77.21 ± 2.65	−1.25	**0.012**
	Inferior	77.31 ± 2.51	78.35 ± 2.62	−1.04	**0.036**
RPE	Superior	12.30 ± 1.18	12.64 ± 1.19	−0.33	0.138
	Inferior	12.65 ± 1.36	12.84 ± 1.35	−0.19	0.460

The values represent mean ± standard deviation. Student’s t-test was used for independent samples. Significant thinning in early POAG are indicated in blue. Significant thickenings in early POAG are indicated in red. Significance is indicated in bold. Mrnfl = macular retinal nerve fiber layer; GCL = Ganglion cell layer; IPL = Inner plexiform layer; INL = Inner nuclear layer; OPL and ONL = combination of outer plexiform layer and outer nuclear layer; PRL = photoreceptor layer; RPE = Retinal pigment epithelium. The difference of means corresponds to OHT-POAG.

## References

[1] Quigley H.A., Broman A.T. (2006). The number of people with glaucoma worldwide in 2010 and 2020. Br. J. Ophthalmol..

[2] Jonas J.B., Aung T., Bourne R.R., Bron A.M., Ritch R., Panda-Jonas S. (2017). Glaucoma. Lancet.

[3] Weinreb R.N., Friedman D.S., Fechtner R.D., Cioffi G.A., Coleman A.L., Girkin C.A., Liebmann J.M., Singh K., Wilson M.R., Wilson R. (2004). Risk assessment in the management of patients with ocular hypertension. Am. J. Ophthalmol..

[4] Curcio C.A., Allen K.A. (1990). Topography of ganglion cells in human retina. J. Comp. Neurol..

[5] Mohammadzadeh V., Fatehi N., Yarmohammadi A., Lee J.W., Sharifipour F., Daneshvar R., Caprioli J., Nouri-Mahdavi K. (2020). Macular Imaging with Optical Coherence Tomography in Glaucoma. Surv. Ophthalmol..

[6] Kolb H., Nelson R.F., Ahnelt P.K., Ortuño-Lizarán I., Cuenca N., Kolb H., Fernandez E., Nelson R. (2020). The Architecture of the Human Fovea. Webvision: The Organization of the Retina and Visual System.

[7] Wang M., Hood D.C., Cho J.S., Ghadiali Q., De Moraes C.G., Zhang X., Ritch R., Liebmann J.M. (2009). Measurement of local retinal ganglion cell layer thickness in patients with glaucoma using frequency-domain optical coherence tomography. Arch. Ophthalmol..

[8] Kotowski J., Folio L.S., Wollstein G., Ishikawa H., Ling Y., Bilonick R.A., Kagemann L., Schuman J.S. (2012). Glaucoma discrimination of segmented cirrus spectral domain optical coherence tomography (SD-OCT) macular scans. Br. J. Ophthalmol..

[9] Kita Y., Kita R., Takeyama A., Anraku A., Tomita G., Goldberg I. (2013). Relationship between macular ganglion cell complex thickness and macular outer retinal thickness: A spectral-domain optical coherence tomography study. Clin. Exp. Ophthalmol..

[10] Pazos M., Dyrda A.A., Biarnés M., Gómez A., Martín C., Mora C., Fatti G., Antón A. (2017). Diagnostic accuracy of spectralis SD OCT automated macular layers segmentation to discriminate normal from early glaucomatous eyes. Ophthalmology.

[11] Cifuentes-Canorea P., Ruiz-Medrano J., Gutierrez-Bonet R., Peña-Garcia P., Saenz-Frances F., Garcia-Feijoo J., Martinez-de-la-Casa J.M. (2018). Analysis of inner and outer retinal layers using spectral domain optical coherence tomography automated segmentation software in ocular hypertensive and glaucoma patients. PLoS ONE.

[12] Unterlauft J.D., Rehak M., Böhm M.R.R., Rauscher F.G. (2018). Analyzing the impact of glaucoma on the macular architecture using spectral-domain optical coherence tomography. PLoS ONE.

[13] Vianna J.R., Butty Z., Torres L.A., Sharpe G.P., Hutchison D.M., Shuba L.M., Nicolela M.T., Chauhan B.C. (2019). Outer retinal layer thickness in patients with glaucoma with horizontal hemifield visual field defects. Br. J. Ophthalmol..

[14] Ishikawa H., Stein D.M., Wollstein G., Beaton S., Fujimoto J.G., Schuman J.S. (2005). Macular segmentation with optical coherence tomography. Investig. Ophthalmol. Vis. Sci..

[15] Fan N., Huang N., Lam D.S., Leung C.K. (2011). Measurement of photoreceptor layer in glaucoma: A spectral-domain optical coherence tomography study. J. Ophthalmol..

[16] García-Medina J.J., Del-Rio-Vellosillo M., Palazón-Cabanes A., Tudela-Molino M., Gómez-Molina C., Guardiola-Fernández A., Villegas-Pérez M.P. (2018). Mapping the thickness changes on retinal layers segmented by spectral-domain optical coherence tomography using the posterior pole program in glaucoma. Arch. Soc. Esp. Oftalmol..

[17] Chen Q., Huang S., Ma Q., Lin H., Pan M., Liu X., Lu F., Shen M. (2017). Ultra-high resolution profiles of macular intra-retinal layer thicknesses and associations with visual field defects in primary open angle glaucoma. Sci. Rep..

[18] Doughty M.J., Zaman M.L. (2000). Human corneal thickness and its impact on intraocular pressure measures: A review and meta-analysis approach. Surv. Ophthalmol..

[19] Hodapp E., Parrish R.K.I.I., Anderson D.R. (1993). Clinical Decisions in Glaucoma.

[20] Lujan B.J., Roorda A., Croskrey J.A., Dubis A.M., Cooper R.F., Bayabo J.K., Duncan J.L., Antony B.J., Carroll J. (2015). Directional optical coherence tomography provides accurate outer nuclear layer and henle fibre layer measurements. Retina.

[21] Sullivan-Mee M., Ruegg C.C., Pensyl D., Halverson K., Qualls C. (2013). Diagnostic precision of retinal nerve fibre layer and macular thickness asymmetry parameters for identifying early primary open-angle glaucoma. Am. J. Ophthalmol..

[22] Ghasia F.F., El-Dairi M., Freedman S.F., Rajani A., Asrani S. (2015). Reproducibility of spectral- domain optical coherence tomography measurements in adult and pediatric glaucoma. J. Glaucoma.

[23] Rolle T., Manerba L., Lanzafame P., Grignolo F.M. (2016). Diagnostic Power of Macular Retinal Thickness Analysis and Structure-Function Relationship in Glaucoma Diagnosis Using SPECTRALIS OCT. Curr. Eye Res..

[24] Kim H.J., Lee S.Y., Park K.H., Kim D.M., Jeoung J.W. (2016). Glaucoma Diagnostic Ability of Layer-by-Layer Segmented Ganglion Cell Complex by Spectral-Domain Optical Coherence Tomography. Investig. Ophthalmol. Vis. Sci..

[25] Michelessi M., Riva I., Martini E., Figus M., Frezzotti P., Agnifili L., Manni G., Quaranta L., Miglior S., Posarelli C. (2019). Macular versus nerve fibre layer versus optic nerve head imaging for diagnosing glaucoma at different stages of the disease: Multicenter Italian Glaucoma Imaging Study. Acta Ophthalmol..

[26] Barua N., Sitaraman C., Goel S., Chakraborti C., Mukherjee S., Parashar H. (2016). Comparison of diagnostic capability of macular ganglion cell complex and retinal nerve fibre layer among primary open angle glaucoma, ocular hypertension, and normal population using Fourier-domain optical coherence tomography and determining their functional correlation in Indian population. Indian J. Ophthalmol..

[27] Edlinger F.S.M., Schrems-Hoesl L.M., Mardin C.Y., Laemmer R., Kruse F.E., Schrems W.A. (2018). Structural changes of macular inner retinal layers in early normal-tension and high-tension glaucoma by spectral-domain optical coherence tomography. Graefes Arch. Clin. Exp. Ophthalmol..

[28] Tsamis E., Bommakanti N.K., Sun A., Thakoor K.A., De Moraes C.G., Hood D.C. (2020). An automated method for assessing topographical structure–function agreement in abnormal glaucomatous regions. Trans. Vis. Sci. Tech..

[29] Kim K.E., Jeoung J.W., Park K.H., Kim D.M., Kim S.H. (2015). Diagnostic classification of macular ganglion cell and retinal nerve fibre layer analysis: Differentiation of false-positives from glaucoma. Ophthalmology.

[30] Wilsey L.J., Reynaud J., Cull G., Burgoyne C.F., Fortune B. (2016). Macular Structure and Function in Nonhuman Primate Experimental Glaucoma. Investig. Ophthalmol. Vis. Sci..

[31] Nork T.M., Ver Hoeve J.N., Poulsen G.L., Nickells R.W., Davis M.D., Weber A.J., Vaegan Sarks S.H., Lemley H.L., Millecchia L.L. (2000). Swelling and loss of photoreceptors in chronic human and experimental glaucomas. Arch. Ophthalmol..

